# The Shape of Water: How Tai Chi and Mental Imagery Effect the Kinematics of a Reach-to-Grasp Movement

**DOI:** 10.3389/fphys.2020.00297

**Published:** 2020-04-09

**Authors:** Luisa Sartori, Andrea Spoto, Matteo Gatti, Elisa Straulino

**Affiliations:** ^1^Dipartimento di Psicologia Generale, Università di Padova, Padua, Italy; ^2^Padova Neuroscience Center, Università di Padova, Padua, Italy; ^3^Università Popolare Wang Academy, Milan, Italy

**Keywords:** Tai Chi, mental imagery, enhanced motor functions, kinematics, motor control

## Abstract

The aim of the present study was to investigate the effect of Tai Chi (TC) and mental imagery (MI) on motor performance. MI is the ability of representing different types of images and it can be improved through constant practice (e.g., of TC). The majority of previous literature has mainly investigated the impact of this mental factor by means of qualitative indexes, whereas studies considering more rigorous measures such as kinematic parameters are rare. In this vein, little is known about how MI can affect reach-to-grasp, one of the most studied models in kinematic research. The present study attempts to fill that gap by investigating the relationship between MI and motor performance in TC, a practice that largely promotes the adoption of mental training. One TC master, four instructors, ten apprentices and fifteen untrained participants were requested to reach toward and grasp an object while mentally representing one out of five different images related to water with an increasing degree of dynamicity and expansion (i.e., still water, flowing water, wave, whirlpool, and opening water flower). Kinematic profiles of movements were recorded by means of six infra-red cameras using a 3-D motion analysis system. We tested whether: (i) focusing on MI during the task would help in optimizing motor efficiency, and (ii) expertise in TC would be reflected in higher flexibility during the task. The results indicate that kinematics is highly sensitive to MI and TC practice. In particular, our main finding suggests a statistically significant general improvement in motor efficiency for the TC group and a beneficial effect for all the participants when focusing on the most expansive image (i.e., opening water flower). Moreover, regression analysis indicates that MI and TC practice make online control more flexible in an experience-based way. These results have important implications for the use of mental imagery and TC in the retraining of motor function in people with physical disabilities.

## Introduction

Tai Chi (TC) is a moving mind-body exercise characterized by circular, slow, and smooth movements that originated in China more than 1200 years ago (Li et al., 2001). It is well-established that the slow tempo facilitates a sensory awareness of the speed, force, trajectory, and execution of movement throughout the practice (Tse and Bailey, 1992). In TC, the body is naturally extended and relaxed, the mind is calm but alert, and body movements are well-coordinated. The practice includes balanced body positions that flow from one to the next and promote mental concentration to improve motor efficiency and flexibility, two aspects that lay the foundation for an optimized motor control (Wahbeh et al., 2008). Recently, a few Chinese Masters (e.g., Wang Zhuanghong, 1931–2008) have restored a classical TC practice that emphasizes mental imagery (MI) rather than motor repetition or aesthetical aspects. In this respect, TC has more to offer than a simple relaxation technique. A deep investigation of its movement principles could furnish a new mind-body perspective able to highlight its impact on kinematic fluidity and to develop new interventions for improving motor control and (re)learning.

Despite a growing interest on the mechanical bases of TC, most of the literature is largely based on qualitative or poorly controlled observations. In particular, the low quality of the research designs, the limitations in the methodology (e.g., inadequate control groups, deficient statistical analyses, no randomized trials) and their great heterogeneity (e.g., huge variability in series of postures usually called “forms”) make comparisons across studies very difficult. Here we adopted an ecologically valid paradigm in conjunction with objective methods and statistics, and two controlled groups of participants covering a full range of expertise levels.

Until recently few studies have focused on the effect of TC by means of 3-D analysis of movement (e.g., Law and Li, 2014). Kinematics is the mathematical description of movement, which is defined in terms of velocity, time, trajectories and acceleration. To our knowledge, no study has examined the relationship between TC and MI during a reach-to-grasp movement. This simple everyday gesture might prove to be an effective experimental task to objectively compare people who perform this practice with untrained participants. Testing a learned TC form would not allow such comparison in a control group, since expertise would play a crucial role. Analyzing the kinematic characteristics of a simple reach-to-grasp task (i.e., an ecologic framework), instead, might lend insight into which aspects of motor control are affected by TC.

Reaching and grasping objects represents a basic gesture that humans perform routinely in a variety of contexts and that requires the coordination of multiple upper extremity segments (Sejnowski, 1998). It is also a movement that has been well characterized experimentally in terms of two functionally coupled components: a transport component, in charge of moving the arm close to the object, and a grip component, responsible for preparing the hand (i.e., pre-shaping) to capture the object (for a review see Castiello, 2005). Both components are sensitive to different characteristics of target object (e.g., object size and spatial location; Jakobson and Goodale, 1991), as well as to the agent’s intention in grasping the object (e.g., grasping a bottle for pouring versus for throwing; Ansuini et al., 2008), so that although the to-be-grasped object remains the same, different kinematic patterns are produced. In particular, a large number of kinematic studies have highlighted that when task complexity requires a careful or difficult positioning of fingertips on the object, a longer deceleration occurs while approaching the target and progressively closing the fingers on it before contact (Marteniuk et al., 1990; Becchio et al., 2008a, b; Sartori et al., 2009). This compensatory strategy allows extra time to make on-line corrections based on visual feedback (i.e., a safety margin; Wing et al., 1986; van Vliet and Sheridan, 2007; Michaelsen et al., 2009). When driving a car, for instance, a longer deceleration is needed in order to precisely stop at the red light if the car is novel or the pilot is inexperienced, otherwise your vehicle may miss the target. The deceleration phase in patients with post-stroke hemiparesis, for instance, is longer than that of healthy subjects (van Vliet and Sheridan, 2007). Once driving is well-performed, instead, the braking will be short, gentle and precise. The same principle applies to reaching and grasping movements, with later deceleration and closing of the hand indicative of a more dexterous movement (Castiello et al., 1992; Becchio et al., 2008a, b). This reflects the fact that a greater part of the movement is centrally programmed (ballistic) and a small amount of time is needed to calibrate fingertip placement on the object (Sartori et al., 2011).

Notably, improved reaching and grasping efficiency is subtended by a decreased muscular activation (Wagner et al., 2007), thereby highlighting a strong relationship between kinematic performance and the muscular effort to perform the task. In particular, since individuals execute hundreds of reach movements throughout a typical day, then it is critical that each repetition of the movement requires only a minimal muscle effort (Wagner et al., 2007).

Taken together, those findings represent an important advancement in the motor control literature as the study of intentions and motor strategies during the performance of a simple motor task provides an indication of their systemic importance. Nowadays, motor execution is no longer seen as a purely mechanical process, dissociated from mental components.

Interestingly, TC Classics from the 18th Century claim that TC entails two levels: (i) building a set of fundamental postures to attain smooth and circular movements; and ii) using mental intent to guide movement. The mind should not directly focus on how to perform a movement. Rather, we should focus on intention and this will (indirectly) move the body (Ying and Chiat, 2013). Moving the external body without involving the mind is not TC (Ying and Chiat, 2013). A central concept of TC is indeed a focused attention on a specific scenery or an image in a realistic visualization (i.e., with real sensations and perceptions) for an extended period of time. Indeed, directing attention to a specific image – instead of the movement itself – is known to facilitate motor performance (Abdollahipour et al., 2015; Schmalzl et al., 2015), and may accelerate motor (re)training by promoting the execution of natural and quasi-automatic movements (Wulf and Lewthwaite, 2010).

The pioneering work on piano playing by Pascual-Leone (2001) has shown that the acquisition of a new skill is characterized by a three-step progression:

Step 1 | *Basic skill acquisition*. Attention is focused on controlling every single movement. “At the beginning, the limbs move slowly, with fluctuating accuracy and speed, and success requires visual, proprioceptive, and auditory feedback.” (Pascual-Leone, 2001, p. 316). A well-known principle in motor control literature is that focusing on proprioceptive information is crucial to improve neuromuscular control (Irrgang and Neri, 2000; Eils and Rosenbaum, 2001). In this respect, TC can be particularly proficient in increasing joint proprioception by providing ideal exercises (e.g., kinetic chain exercises that enhance conscious awareness of joint position and movement; Irrgang and Neri, 2000; Cerulli et al., 2001; Hong and Li, 2007).

Step 2 | *Motor Efficiency*. Once the basic skill is acquired, muscular effort is reduced and attention can be shifted to mastering the technique. “Eventually, each single movement is refined, the different movements chained into the proper sequence with the desired timing, … and a fluency of all movement developed” (Pascual-Leone, 2001, p. 316). Motor Efficiency is measurable by dexterity in performance and decreased muscular effort. In this regard, kinematics can offer an indirect measure of muscular patterns, as revealed by a recent study combining kinematic and EMG measures during the execution of reach-to-grasp actions. In particular, Betti et al. (2018) showed that a short phase of hand closing on the target – indexed by a late grip aperture – was correlated with a decreased activation of hand muscles (Betti et al., 2018). More in general, combined EMG-kinematic studies associate reaching efficiency with reduced wrist velocity and deceleration (van Vliet and Sheridan, 2007; Wagner et al., 2007), while good accuracy and later closing of the hand are signs of grasping dexterity (Castiello et al., 1992; Becchio et al., 2008a, b).

Step 3 | *Mental integration*. Once the technique is refined, all the cognitive resources can be devoted to mental aspects. “Only then can the pianist shift his or her attentional focus away from the mechanical details of the performance toward the emotional content of the task.” This aspect is in line with the concept of “flow” (Csikszentmihalyi, 1991), which entails the execution of natural and flawless movements whilst the mind is fully concentrated.

In this framework, the TC classics specifically highlight a fourth level, that can be trained from Step 2 on out:

Step 4 | *Motor Flexibility*. Intention moves the body and this leads to a new achievement in terms of performance: Motor Flexibility, defined as the ability to modulate even an automatized movement (e.g., when walking on a slippery surface). A flexible motor control would allow an athlete to compensate for severe injuries and still perform at the highest levels, overcoming a particular impairment at a given moment. The ability to integrate more options at a time is a trademark of Motor Flexibility, and kinematics has been extensively used to track this feature of the on-line motor control system. In particular, Trajectory Deviation has been classically used to reveal the concomitant presence of different motor plans (Chieffi et al., 1993; Howard and Tipper, 1997; Sartori et al., 2009). Here we considered Motor Flexibility as the ability to modulate the trajectory path of an automatized movement by focusing on a highly dynamic image at a given moment. Moreover, capitalizing on the seminal work by Xu et al. (2003) on kinematics and EMG analysis, we computed the Range (i.e., difference between the maximum and the minimum values across conditions) for all kinematic parameters as an index of motor variability, associated with Motor Flexibility. In this regard, we should distinguish between the concepts of “External” variability (i.e., induced by an external agent or the context) and “Internal” variability (i.e., inherent in the motor system when performing a task). External variability can promote adaptability to novel or different contexts (Merbah and Meulemans, 2011), as highlighted in the Motor Schema Theory (Schmidt, 1975). A generalized motor program consists of an abstract memory structure apt to guide a range of movements (e.g., reach-to-grasp actions; Schmidt, 1975). These actions are subserved by a Schema (i.e., a rule developed by practice) which describes a relationship between the outcome of a program and the chosen parameters. Interestingly, increasing variability in experiences can lead to increased generalization, yielding therefore superior performance in terms of movement adaptation. Internal variability, then, is the repertoire of motor Schema from which we can select to adapt successfully to changes. In this regard, a classic example are the hammer trajectories of expert blacksmiths who can hit the target with a functional variability (Bernshteǐn, 1967). In highly skilled movements, internal variability should not be confused with lack of consistency or variable error (e.g., a large spread in the distribution of an archer’s arrows or a Parkinson’s tremor). It is often assumed that successful outcomes can only arise from high consistency in movement execution (e.g., low variability in the kinematics of the hand). But this is not necessarily the case. Recent research in motor control has shown that a certain degree of internal variability (i.e., motor flexibility) is required for optimal motor performance (Harbourne and Stergiou, 2009; Ranganathan and Newell, 2013).

Despite the emerging body of research indicating a link between MI and modulation of performance, further research is required. In particular, it is currently unknown whether the combined use of TC and MI may produce more flexible motor control. Furthermore, it is possible that expertise may differentially modulate this effect, but this has not yet been tested.

Mental imagery is one of the most characteristic aspects of human thought. It is conceived as mental representations of events or stimuli in the absence of sensory inputs from the external world (Palmiero et al., 2019). TC training is typically rich in images such as “move like a river or light as a cloud” that guide practitioners toward specific kinesthetic states. A considerable amount of research in psychology has focused on techniques such as MI in order to draw attention away from the everyday mind flow, reach an attentive state and attain a better performance (Abdollahipour et al., 2015; Schmalzl et al., 2015).

The aim of the present study was to characterize how a reach-to-grasp action (representative of everyday motor performance) is influenced by TC training that incorporates a significant degree of MI. We adopted a simple motor task that is performed hundreds of times every day to consider both TC performers and untrained participants, therefore minimizing the confounding variable of movement expertise. Expertise is generally defined as the highest level of performance on a specific task or within a specific domain (Bourne et al., 2014). Testing different groups of people on this task allowed us to exclude possible effects caused by different sport practices.

We enrolled a group of TC practitioners and a group of control participants. Specifically, we applied a reach-to-grasp paradigm to assess the effect of five mental images while grasping and lifting an object between the thumb and palm, i.e., a whole-hand grasp. In this vein, we chose a school of TC that specifically focuses on mental concentration and imagery from the beginner level, rather than introducing it only at the advanced level. In this regard, motor repetition is not predominant with respect to the mental component of movement. The TC master representing this MI approach carefully selected three mental images related to water: flowing water, wave, and whirlpool. These images imply a movement of water with an increasing degree of dynamicity. He then added a fourth item: opening water flower, an image that implies an expanding movement and can produce a feeling of marvel. Emotions involve specific peripheral physiological responses, which can enable emotion-specific actions (Zhang and Keltner, 2016). Notably, all these images were novel and had not previously been learned by his practitioners. In order to set a baseline value we devised a neutral fifth image, still water, pertaining to the water context but not implying any movement. The rationale for adopting a neutral image instead of giving no images at all was to exclude the possibility that participants adopted an (uncontrolled) image on their own.

Finally, since TC is thought to determine better mental-attentional vigilance (Kim et al., 2016) we specifically tested this aspect by means of the Continuous Performance Test (CPT), a widely used measure of sustained attention (Hsieh et al., 2005). Testing this variable was aimed at verifying possible correlations with TC practice.

We hypothesized that focusing on MI during the task would influence prehensile kinematics, in particular for the group already trained at adopting MI strategies. If the combined use of mental images during TC training develops the ability to perform (i) efficient and (ii) flexible movements, then it should affect also automatized movements such as reach-to-grasp actions. In particular, we expected a well-calibrated and timely grip aperture (Jeannerod et al., 1995) in both the TC and Non-TC groups, due to the fact that the task is simple and highly automatized. However, if TC participants are more able to take advantage of mental images than the control group, this accuracy should be achieved with less cost. Specifically, a greater (i) Motor Efficiency should be revealed by a lower wrist velocity and deceleration during the reaching phase, and by a firm closure of the fingers around the object without safety margins. As concerns (ii) Motor Flexibility, we expected an enhanced trajectory deviation associated with the more dynamic image in the TC compared to the Non-TC group. Moreover, we hypothesized a link between TC expertise and motor variability (i.e., the Range of movement kinematics). Motor Flexibility should be reflected in highly consistent movements within conditions, and highly variable movements across conditions, being dependent on a given image at a given time. Lastly, we hypothesized that TC expertise could also produce a better performance on sustained attention.

## Materials and Methods

### Ethics Statement

The experiments were approved by the Ethics Committee of the University of Padua (No 2687), in accordance with the Declaration of Helsinki (Sixth revision, 2008). All participants signed written informed consent prior to the beginning of their experimental session.

### Participants

**FIGURE 1:**
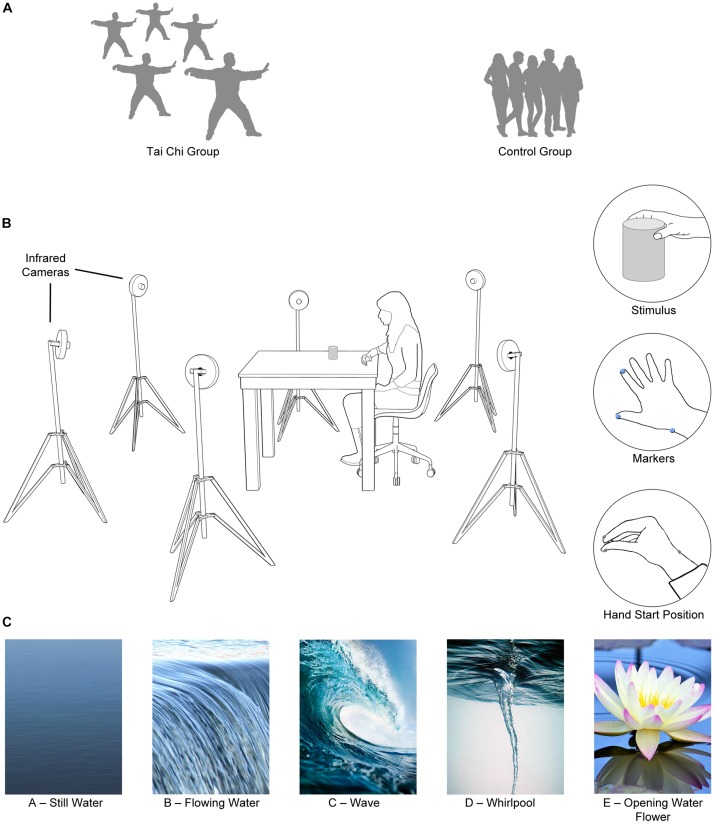
*Set up and procedure*. **(A)** Two groups of participants were recruited: a TC and a Control Group. **(B)** A 3D-Optoelectronic SMART-D system was used to track the kinematics of the participant’s right upper limb by means of six video cameras. Infrared reflective markers were taped to the following points: thumb, index finger, and wrist to measure the grasp and reach component of the movement. Participant’s hand was positioned with the thumb and the index finger in opposition on a starting pad located at 35 cm from the cylinder they had to grasp after the Go signal. **(C)** During the reach-to-grasp task, participants were instructed to focus on a different mental image for each condition (Still Water, Flowing Water, Wave, Whirlpool or Opening Water Flower).

Thirty participants took part in the experiment: 15 TC practitioners (with different expertise levels) and 15 untrained participants. In particular, one élite TC Master (age: 60; years of experience: 40), four TC Instructors (age range: 40–54; mean of years’ experience: 10; 3 males and 1 female) and ten TC Apprentices (age range: 28–71; mean of years’ experience: 6; 6 males and 4 females) from a local TC School, and fifteen Control Participants (age range: 21–35; 5 male and 10 female) from a local gym without any TC experience, were recruited at the Neuroscience of Movement Laboratory (NeMo) (Figure 1A). All the participants were right-handed (Briggs and Nebes, 1975) and had normal or corrected-to-normal vision. They were naïve to the experimental design and study purpose.

### Stimulus

The stimulus was a wooden cylinder (weight 150 g, height 11 cm, diameter 7.5 cm) located at 35 cm from the hand start position (Figure 1B) in all conditions.

### Procedure

Before starting the experimental session, all participants performed the CPT on a computer to assess their sustained attention. A time series of letters (from A to Z) were randomly presented on a monitor and participants were instructed to press a switch button in response to each letter, except for a critical stimulus. The critical stimulus was a particular single stimulus out of the available set (i.e., the letter “X”). Participants were instructed to perform the task as correctly as possible. Letters were presented on a 5 cm square matrix. The inter-stimulus interval was 800 ms and the stimulus duration was 200 ms. The available response time was 700 ms. The CPT test began with 2 min of practicing and it lasted 10 min. For the reach-to-grasp trials, the participants then sat on a height adjustable chair in front of a working surface with elbow and wrist on the table (90 × 90 cm). All participants were tested individually. Before each trial, the right hand of each participant was resting on a starting pad with the thumb and the index finger gently in opposition (Figure 1B). The task was to reach toward and grasp the cylinder, while focusing on a mental image. The motor task was kept deliberately simple so as to facilitate MI with open eyes. The participants were requested to start the action after a go-signal was delivered and they were tested in five randomized experimental conditions (Figure 1C), each corresponding to a different mental image: still water (condition A), flowing water (condition B), wave (condition C), whirlpool (condition D) and opening water flower (condition E). Each participant performed ten trials for each condition, for a total of 50 trials.

### Apparatus

Six infrared cameras (sampling rate 140 Hz) detecting three infrared reflective markers (6 mm diameter) were placed in a semicircle at a distance of 1–1.2 meters from the room’s center. The movements were recorded using a SMART motion analysis system (Bioengineering Technology and Systems [B| T| S]). Cameras captured the movements of the markers in 3D space (Figure 1B). The coordinates of the markers were reconstructed with an accuracy of 0.2 mm over the field of view. The standard deviation of the reconstruction error was 0.2 mm for the vertical (Y) axis and 0.3 mm for the two horizontal (X and Z) ones. In Jeannerod (1981) described two major components for prehensile behavior: the transport and the grasp components. The transport component brings the hand close to the object. The grasp component is concerned with finger pre-shaping during transport and finger closing around the object. Two markers were then placed on thumb (ulnar side of the nail) and index finger (radial side of the nail) to measure the grasping component of the action. One marker was taped on the wrist (dorsodistal aspect of the radial styloid process) to measure the reaching component of the action.

### Data Analysis

Following kinematic data collection, each trial was individually checked for correct marker identification and then run through a low-pass Butterworth filter with a 6 Hz cutoff. The SMART-D Tracker software package (Bioengineering Technology and Systems, B|T| S) was employed to reconstruct the 3-D marker positions as a function of time. One participant was eliminated from the data set due to a technical problem. We selected a set of standard measures classically reported in the literature for reach-to-grasp tasks (Wing et al., 1986; Castiello et al., 1992; Gentilucci et al., 2003), possibly enabling a productive comparison of results across participants (TC practitioners, No-TC) and across experiments. We first computed movement time as the temporal interval between movement onset (i.e., the first time point at which the wrist velocity crossed a 5 mm/sec threshold and remained above it for longer than 500 ms) and time of grip offset, when fingertips made contact with the object (i.e., the time at which the grip closing velocity dropped below the 5 mm/s threshold; Chinellato et al., 2015). Then the following indexes were measured:

• Maximum Wrist Velocity (MWV, the 3D resultant peak of wrist velocity);
• Maximum Wrist Deceleration (MWDec, the maximum deceleration of the 3-D coordinates of the wrist);
• Maximum Trajectory Deviation (MTD, the maximum deviation of the 3-D coordinates of the wrist from the ideal line linking the starting position with the end position);
• Maximum Grip Aperture (MGA, the maximum radial distance reached by the 3-D coordinates of the thumb and index finger);
• Maximum Grip Closing Velocity (MGCV, the maximum velocity of the 3-D coordinates of the thumb and index finger during hand closing);

The temporal peaks were then normalized with respect to movement time, so that individual speed differences were accounted for:

• Time to Maximum Wrist Velocity (TMWV, the proportion of time at which wrist velocity reached its peak from movement onset);
• Time to Maximum Wrist Deceleration (TMWD, the proportion of time at which wrist deceleration reached its minimum peak from movement onset);
• Time to Maximum Trajectory Deviation (TMTD, the proportion of time at which the maximum deviation of the 3D coordinates of the wrist occurred from movement onset);
• Time to Maximum Grip Aperture (TMGA, the proportion of time at which thumb and index finger reached a maximum distance, calculated from movement onset);
• Time to Maximum Grip Closing Velocity (TMGCV, the proportion of time at which thumb and index finger reached a maximum closing velocity from movement onset);

For each variable the Range (i.e., difference between the maximum and the minimum values across conditions; Woodbury, 2001) was computed as an index of Motor Flexibility (for a similar approach see Xu et al., 2003) and was used as the dependent variable in a regression analysis with years of TC practice. Finally, we tested the difference between the two groups on the d’ (Heeger, 1997; Macmillan and Creelman, 2004) of the CPT. According to the Signal Detection Theory, the d’ (sensitivity) is a measure of the subject’s ability to discriminate a signal (here, the letter “X” appearing in a very few trials) from the background noise (i.e., all the other letters). A higher d’ indicates better processing capabilities. In the present experiment, a better ability to discriminate the crucial letter for a long time would reflect a good sustained attention.

### Statistical Analyses

Behavioral data were analyzed using the jamovi 0.9.6.9 statistical software (The jamovi project, 2019). Data analysis was divided into two main parts: the first one was aimed at testing the effect of belonging to the TC group on the behavioral variables related to the reach-to-grasp task in the different sessions of the experiment (i.e., eliciting different mental images); the second one was aimed at determining the role of TC practice focused on MI in predicting motor flexibility. The first part of the analysis consisted in fitting Linear Mixed Effect Models having the five Sessions (A, B, C, D, and E) as within fixed effects, the two Groups (TC and No-TC) as between fixed effects, and Individuals as random effects. Such models, including contrasts and *post hoc* comparisons with Bonferroni correction, were fitted for each of the kinematic variables. All the models were fitted using the General Analyses for Linear Models of the jamovi software (Gallucci, 2019). The second part of the analysis consisted in fitting a number of linear regression models on the same dependent variables, having as predictor the number of years of TC practice. These analyses were conducted using the Companion to Applied Regression V.3 (Fox and Weisberg, 2018) and the Estimated Marginal Means version 1.4.1 (Lenth et al., 2019) packages of the statistical software R (R Core Team, 2018), implemented in jamovi. As concerns the CPT, we used the proportion of hits (i.e., proportion of correct detection) and false alarms (i.e., proportion of stimuli reported when not present) to calculate the d’. Before the d’ calculation, the hit and false alarm proportions were corrected by adding 0.5 to both the number of hits and the number of false alarms and adding 1 to both the number of signal trials and the number of noise trials to avoid indeterminate values (loglinear approach; Hautus, 1995). *d*’ values were then submitted to an independent samples *t*-test.

## Results

### Linear Mixed Effect Models

Means and Standard Deviations for each kinematic parameter, experimental conditions and group are reported in Table 1. Significant fixed effects emerged out of several models. Moreover, planned contrasts highlighted, for two variables, different behavioral patterns in Session E compared with all the other ones.

**TABLE 1:** Means and Standard Deviations in brackets for each kinematic parameter, experimental conditions and group.

	A		B		C		D		E	
	Control	TC	Control	TC	Control	TC	Control	TC	Control	TC
MT	1.25(±0.26)	1.92(±0.64)	1.25(±0.29)	1.90(±0.58)	1.33(±0.33)	1.88(±0.55)	1.24(±0.25)	1.78(±0.53)	1.35(±0.29)	2.03(±0.70)
MWV	0.66(±0.13)	0.47(±0.13)	0.66(±0.11)	0.49(±0.14)	0.64(±0.13)	0.49(±0.13)	0.66(±0.12)	0.48(±0.12)	0.61(±0.11)	0.46(±0.14)
MWDec	2.18(±0.73)	1.22(±0.55)	2.11(±0.62)	1.35(±0.63)	2.11(±0.64)	1.33(±0.59)	2.16(±0.72)	1.34(±0.53)	1.91(±0.61)	1.2(±0.59)
MWDev	22.25(±7.93)	28.02(±10.66)	22.94(±8.90)	33.21(±17.53)	22.12(±7.07)	29.5(±16.77)	20.62(±8.58)	38.68(±18.29)	20.77(±7.42)	29.97(±12.86)
MA	129.19(±8.03)	126.9(±8.58)	129.46(±8.50)	125.74(±7.28)	129.05(±5.68)	126.5(±7.96)	129.86(±7.78)	125.11(±8.14)	129.76(±7.44)	127.17(±8.47)
MGCV	0.2(±0.07)	0.11(±0.04)	0.19(±0.06)	0.11(±0.03)	0.2(±0.05)	0.13(±0.06)	0.21(±0.08)	0.11(±0.06)	0.19(±0.06)	0.1(±0.04)
TMWV	0.35(±0.05)	0.35(±0.05)	0.37(±0.06)	0.37(±0.06)	0.35(±0.04)	0.38(±0.07)	0.37(±0.05)	0.37(±0.05)	0.36(±0.07)	0.35(±0.05)
TMWDec	0.54(±0.09)	0.51(±0.1)	0.57(±0.1)	0.54(±0.14)	0.57(±0.1)	0.57(±0.14)	0.56(±0.09)	0.57(±0.13)	0.55(±0.09)	0.53(±0.1)
TMWDev	0.47(±0.09)	0.42(±0.07)	0.45(±0.1)	0.43(±0.07)	0.48(±0.09)	0.42(±0.06)	0.46(±0.07)	0.42(±0.05)	0.47(±0.09)	0.43(±0.06)
TMA	0.66(±0.05)	0.7(±0.09)	0.66(±0.04)	0.72(±0.08)	0.68(±0.03)	0.71(±0.08)	0.67(±0.04)	0.72(±0.08)	0.67(±0.05)	0.7(±0.06)
TMGCV	0.84(±0.02)	0.86(±0.04)	0.83(±0.03)	0.87(±0.04)	0.84(±0.04)	0.87(±0.03)	0.84(±0.02)	0.86(±0.04)	0.84(±0.03)	0.87(±0.04)

#### Movement Time

The analysis showed significant main effects of Group (*F*_1__.__27_ = 13.74; *p* < 0.001) and Session (*F*_4__.__108_ = 3.60; *p* = 0.009). The TC group presented higher Movement Time (*M* = 1.90s; SD = 0.59) compared with the Non-TC control group (*M* = 1.29s; SD = 0.28). *Post hoc* comparisons showed that the TC group had significantly higher values with respect to Non-TC control group in Session A (*t*_34__.__85_ = −3.66; *p* = 0.037), B (*t*_34__.__85_ = −3.79; *p* = 0.026), and E (*t*_34__.__85_ = −3.82; *p* = 0.024).

#### Maximum Grip Closing Velocity

**FIGURE 2:**
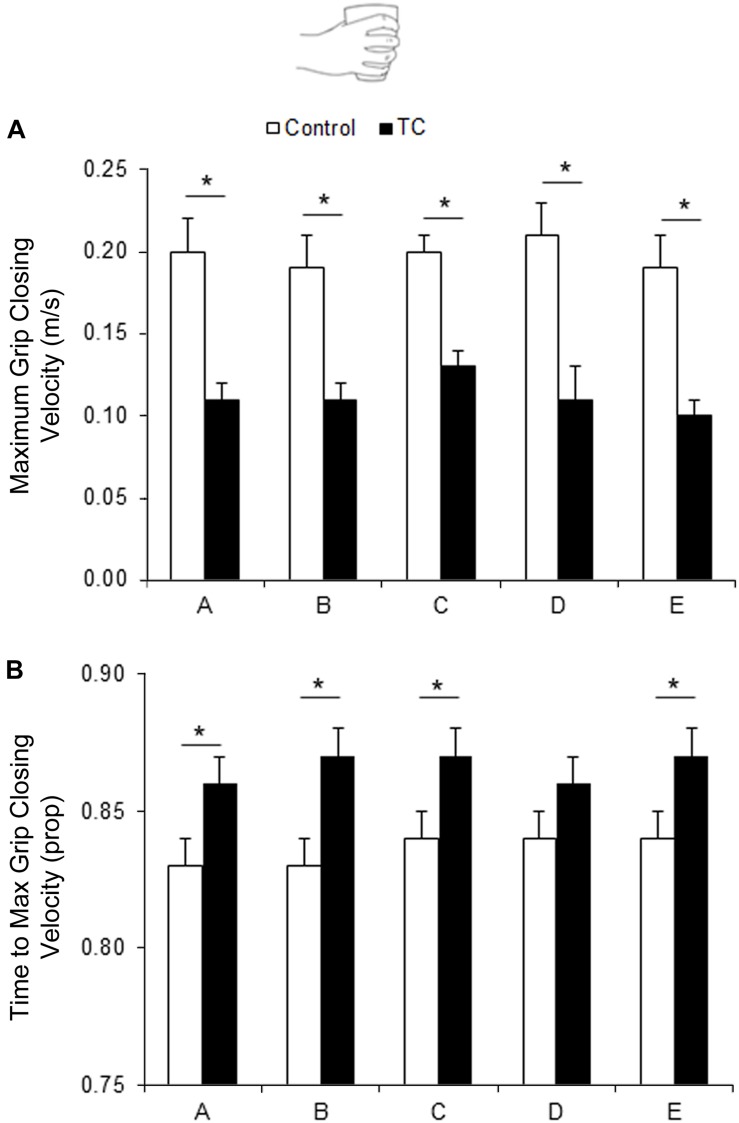
Graphical representation of the mean values for **(A)** the Maximum Grip Closing Velocity and **(B)** the Time to Maximum Grip Closing Velocity. Bars represent SD of the mean. The spatial and temporal components of Grip Closing Velocity indicate that the Finalization Phase of the movement in TC group was characterized by a more careful approach on the object, just before contact. *p < 0.01.

A significant Group main effect was observed (*F*_1__.__27_ = 23.33; *p* < 0.001), with the TC group having lower values (*M* = 0.11 m/s; SD = 0.05) compared to the Non-TC control group (*M* = 0.20 m/s; SD = 0.06; see Figure 2A). The *post hoc* comparisons with Bonferroni correction highlighted that the TC group presented significantly lower values with respect to Non-TC control group in Sessions A (*t*_44__.__50_ = −4.46; *p* = 0.002), B (*t*_44__.__50_ = −3.84; *p* = 0.017), C (*t*_44__.__50_ = −3.68; *p* = 0.028), D (*t*_44__.__50_ = −4.78; *p* < 0.001), and E (*t*_34__.__85_ = −4.43; *p* = 0.003).

### Maximum Wrist Velocity

A significant main effect of the group was found (*F*_1__.__27_ = 15.54; *p* < 0.001), with the TC Group presenting lower values (*M* = 0.49 m/s; SD = 0.13) than the Non-TC control group (*M* = 0.65 m/s; SD = 0.12). *Post hoc* comparisons showed a significant difference between the two groups in Session A (*t*_40__.__80_ = 4.03; *p* = 0.011), B (*t*_40__.__80_ = 3.64; *p* = 0.034), and D (*t*_40__.__80_ = 3.74; *p* = 0.026). Interestingly, the planned contrasts for the Session effect highlighted a significant difference between the values observed for Session E and all the remaining sessions (*t*_108_ = −2.88; *p* = 0.005). More precisely, the values observed for Session E were significantly lower than the remaining ones.

### Maximum Wrist Deceleration

A significant Group effect (*F*_1__.__27_ = 14.48; *p* < 0.001) indicated a significantly smaller deceleration in the TC group (*M* = 1.29 m/s^2^; *SD* = 0.57) than in the Non-TC control group (*M* = 2.09 m/s^2^; SD = 0.65). The contrast indicated a significant difference between the values observed for Session E and all the remaining sessions (*t*_108_ = 2.88; *p* = 0.005), with the former smaller than the others.

### Time of Maximum Grip Closing Velocity

A significant effect of Group was observed (*F*_1__.__27_ = 6.38; *p* = 0.018), with the TC group presenting a later peak (*M* = 0.87; SD = 0.04) than the Non-TC control group (*M* = 0.84; SD = 0.03; see Figure 2B). The *post hoc* comparisons with Bonferroni correction highlighted that the TC group presented significantly more delayed values with respect to Non-TC control group in Sessions A (*t*_44__.__50_ = −3.79; *p* = 0.026), B (*t*_44__.__50_ = −4.56; *p* = 0.004), C (*t*_44__.__50_ = −3.78; *p* = 0.035), and E (*t*_34__.__85_ = −4.03; *p* = 0.010; see Figure 2B).

#### Time of Maximum Wrist Velocity

A significant main effect for the Session was found (*F*_4__.__108_ = 2.76; *p* = 0.031). The *post hoc* analysis did not highlighted any significant differences.

#### Maximum Trajectory Deviation

A significant interaction effect between Group and Session was found (*F*_4__.__108_ = 3.33; *p* = 0.013), indicating differences between the two groups which varied across sessions. *Post hoc* comparisons found a significant difference between the two groups for Session D (*t*_46__.__70_ = −3.87; *p* = 0.015), with the Tai-chi group presenting higher values of right deviation (*M* = 31.90 mm; SD = 15.70) than the Non-TC control group (*M* = 21.70 mm; SD = 7.82).

No significant effects were found with respect to the variables: Time to Maximum Trajectory Deviation (Group: *p* = 0.118. Session: *p* = 0.891. Interaction: *p* = 0.815); Time to Maximum Wrist Deceleration (Group: *p* = 0.701. Session: *p* = 0.061. Interaction: *p* = 0.819); Maximum Grip Aperture (Group: *p* = 0.238. Session: *p* = 0.864. Interaction: *p* = 0.696); Time to Maximum Grip Aperture (Group: *p* = 0.060. Session: *p* = 0.526. Interaction: *p* = 0.345).

A final result refers to the statistically significant difference between the TC and the Non-TC control group on the CPT as indexed by the d’ (*t*_24_ = 2.47; *p* = 0.021, Cohen’s *d* = 0.95). Participants of the TC group presented higher values (*M* = 0.98; SD = 0.82) compared to participants of the control group (*M* = 0.01; SD = 1.17), thus indicating a better sustained attention. In particular, the average Hit score (i.e., number of correct guesses) was 24 vs. 19 for the TC compared to the Non-TC control group, respectively. And the False Alarm score (i.e., number of incorrect guesses) was 12 vs. 17 for the TC with respect to the control group, respectively.

### Linear Regression Models

**FIGURE 3:**
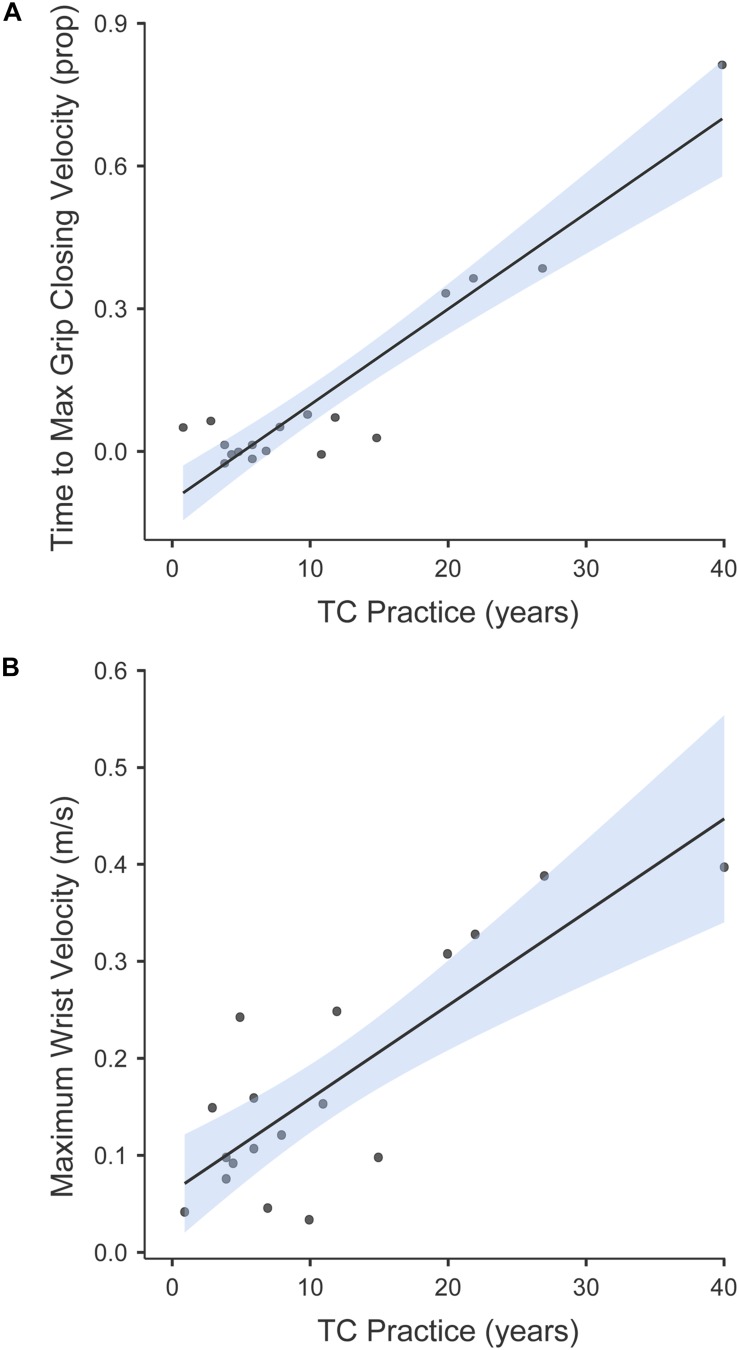
Graphical representation of the regression on Range values for **(A)** the Time to Maximum Grip Closing Velocity and **(B)** the Maximum Wrist Velocity, having as predictor the number of years of TC practice. Expertise was found to predict motor flexibility, with more years of practice predictive of a larger Range of velocity patterns, at the level of both the reaching and grasping components.

The second part of the analysis was aimed at investigating the role of TC practice in predicting the motor flexibility, as described by the Range (i.e., delta for each behavioral datum, between the highest and the lower values across different sessions; see Table 2). This was done by fitting a number of linear regression models having as predictor the number of years of supervised teaching – with a focus on MI, and the delta variables as dependent. Two kinematic deltas were predicted by the number of years of TC practice, namely the Time to Maximum Grip Closing Velocity (β = 0.92; *F*_1__.__13_ = 69.32; *p* < 0.001; *R*^2^ = 0.84; Figure 3A) and the Maximum Wrist Velocity (β = 0.74; *F*_1__.__13_ = 15.43; *p* = 0.002; *R*^2^ = 0.54; Figure 3B).

**TABLE 2:** Means and Standard Deviations for Range of each kinematic parameter.

Range	Mean	SD
MT	0.52	0.35
MWV	0.14	0.10
MWDec	0.48	0.28
MWDev	17.97	12.81
MA	8.06	4.41
MGCV	0.06	0.04
TMWV	0.08	0.04
TMWDec	0.17	0.09
TMWDev	0.10	0.06
TMA	0.09	0.06
TMGCV	0.10	0.20

## Discussion

The primary aim of this experiment was to investigate whether a reach-to-grasp action was influenced by TC training based on MI in a TC group with respect to an untrained group. The most important findings were that the combination of TC and MI was indeed effective in accurately accomplishing the task with significantly less kinematic costs. In line with our prediction, results revealed that TC can influence reach-to-grasp actions, one of the most automatized movements of our everyday life, by adding (i) Efficiency and (ii) Flexibility. Concerning (i) Motor Efficiency, the TC group showed lower wrist velocity and deceleration while approaching the object, consistent with them moving more slowly, indicating the ability to carefully land the arm without the muscular effort of a sudden stop. The grasping component of the movement, moreover, was characterized by a gentler and later closing of the hand while approaching the object – in TC vs. No-TC group, as indicated by the Time and Amplitude of Maximum Grip Closing Velocity. This indicates that there was no need for extra time (i.e., a safety margin) to correctly calibrate fingertip placement on the object. Taken together, these results indicate that a combined training of TC and MI can affect reach-to-grasp kinematics of daily movements performed dozens of times in our everyday life. In particular, it develops the ability to gradually approach objects and to firmly finalize the grasping movement. This, in turns, allows for a more efficient performance. Interestingly, while task accuracy remained constant in both groups (i.e., the scaling of grip aperture was timely and well calibrated to the object size; Jeannerod et al., 1995; Michaelsen et al., 2009), only the TC group showed an enhanced level of (ii) Motor Flexibility, as indicated by a larger deviation of movement trajectory specific for the image characterized by the greatest degree of dynamicity (i.e., “whirlpool,” Session D). Deviations of movement trajectory from an ideal line linking the hand starting position with the object position are used in kinematical literature to unveil concurrent motor programs and flexible reorganization of movement aiming at permitting a range of actions dictated by the environment (e.g., Tipper et al., 1998). Activating parallel representations of all the objects and action possibilities in a given context allows individuals to better navigate the space (e.g., grasping an object without bumping other objects close to the target). TC practice, for instance, teaches you to move as if you were walking on ice, so that once a sudden event occurs, you can always reach stability. In TC terminology, this is termed reversibility principle and it closely refers to flexibility as a prerequisite. Moreover, the TC group showed a larger range of Internal variability across – not within – conditions (i.e., between different images) compared to the Non-TC group. This outcome was present at the level of both the reaching and grasping components (i.e., Maximum Wrist Velocity and Time to Maximum Grip Closing Velocity, respectively). The Feldenkrais Method (Feldenkrais, 1985) of movement awareness holds that functional movements are reversible. When performing a “reversible movement” the mover is not only committed to continue on a trajectory, but can stop, start, or change direction at any time. People normally violate this ideal: it is quite common, for instance, to collide with someone who steps in your path without warning. A movement that can change its mind, instead, might stop midstream, change direction, or continue. The behavioral flexibility of intentional actions can serve as a standard measure of the level of organization, or quality, of the action (Feldenkrais, 1985). According to the Reversibility principle, performing a flexible movement is more adaptive since it permits responses to sudden perturbations in the context (i.e., unexpected changes that occur while we are acting; Haggard, 1994) and to take into account other potential targets. Although a reaching movement seems unequivocally directed to a target object, it is not impermeable to the presence of other objects in proximity (i.e., distractors; Sartori et al., 2012). Evidence suggests that the kinematics of a reach-to-grasp action integrates the motor features of all the objects within a peripersonal space, which might become potential targets. In this vein, developing the skill to activate and maintain multiple motor options for different types of prehension (i.e., motor flexibility) is another strong purpose of TC training. These findings confirm and extend previous literature (e.g., Hong and Li, 2007), by showing that the impact of TC on motor efficiency and flexibility is directly related to its kinematics characteristics. Recently, the World Health Organization has included TC under the heading of “Traditional and Complementary Medicine,” aiming at situating this sector within the national health system of different countries worldwide (WHO, 2013). In this respect, a growing body of clinical research has focused on the efficacy and safety of TC, but little attention has been devoted to evaluating “why” TC is effective. A growing body of research is therefore attempting to precisely test how this practice works. Understanding the mechanisms of this technique in an objective and unbiased way will open new possibilities for the adoption of TC in health care. The future goal is to design stringent paradigms that might allow comparison of findings across experiments.

### Embodied Mental Imagery

A large number of studies have examined both short intense TC trainings (e.g., Gatts and Woollacott, 2007; Huang et al., 2011) and long term effects of TC (Xu et al., 2004; Li et al., 2005; Li, 2014; Manor et al., 2014; Song et al., 2014), but only a few have examined the combination of TC and MI. In a recent study by Alsubiheen et al. (2015), the effect of TC exercise combined with a focus on MI was assessed in terms of enhanced balance control, aimed at restoring some impaired functions due to aging and/or diabetes. The findings of the current experiment extend this literature by providing the first evidence that focusing on a highly dynamic image increased the amplitude of trajectory deviation in a reaching task, which is consistent with a positive impact on motor flexibility. The rationale for adopting “water” as a leading principle in this form of TC is hence evident and straightforward: water has no shape, since it can easily take the container’s shape. In this vein, water is resilient and has potentially infinite shapes, being adaptable to every context. As a last point, water is a natural element and the restorative benefits of nature have been well known for some years (Kaplan, 1995). In the present experiment, adopting a natural image implying an expanding movement with a positive connotation (i.e., the opening water flower, Session E) produced a slowing down of wrist velocity and deceleration – a measure associated with decreased mechanical effort (van Vliet and Sheridan, 2007; Wagner et al., 2007), in both the trained and the untrained group. The combination of TC and MI might thereby be useful in optimizing the retraining of motor function in people with physical disabilities (Dickstein and Deutsch, 2007; Malouin and Richards, 2010). Mental practice becomes relevant especially in persons who do not have the possibility to engage in motor activity, being an appropriate supplement to physical training. For instance, in sub-acute stroke patients with severe motor impairments (Pichiorri et al., 2015), in patients with chronically relapsing diseases of the musculoskeletal system, or in athletes during phases of immobility due to sport concussions (Wolf et al., 1997; Shapira et al., 2001; Xu et al., 2003).

Crucially, objectively quantifying movement kinematics is an important start, but Efficiency and Flexibility should be addressed in a more direct way. This could be considered a pioneering study in that it paves the way for future investigations in which context perturbations or paradigms with target distractors will specifically challenge individual’s motor flexibility. Additional studies should use additional tools such as EMG of upper extremity muscles (e.g., wrist flexor muscles) to provide standardized indexes of motor efficiency and to get a better understanding of motor unit recruitment. Another critical point that was beyond the scope of the present study but will deserve future investigation is the role of individual differences in imagery ability, that is the individual’s capability of forming vivid, controllable images (Palmiero et al., 2019). MI is a form of visual experience triggered internally, from memory, but it shares with perception similar patterns of activation in visual, parietal and frontal cortex (Dijkstra et al., 2019). This similarity is modulated by the vividness of the visual imagery mechanism. In fact, individual differences in creating vivid mental images are positively correlated with the grade of neural overlap (Albers et al., 2013). Interestingly, high-vividness imagers in the motor domain greatly benefit from mental practice with respect to low-vividness imagers (Isaac, 1992). Individual differences in imagery abilities have been classically explored using self-report questionnaires (e.g., Marks, 1973). A new approach to assess vividness differences in the motor domain could be the innovative integration of 3-D analysis of movement during fMRI studies (e.g., Di Bono et al., 2017).

### Tai Chi and Expertise

This study also attempted to clarify whether MI and motor performance might both benefit from constant TC training. Expertise was found to predict Motor Flexibility, with more years of practice predictive of a larger range of motor patterns on both the reaching (wrist velocity) and the grasping (hand closing) components. In addition, although not the main aim of this experiment, it is noteworthy that the TC group surpassed the control group in the CPT, a test aimed at assessing sustained attention. This finding is consistent with the well-established effect that MI helps focusing attention and develops the ability to keep the focus for a long term (Abdollahipour et al., 2015; Schmalzl et al., 2015).

According to Searle (1983), two types of intentions guide our actions: “prior intentions” are the initial representation of the action goal, whereas “intentions-in-action” shape the action, guiding and monitoring it until completion. Intentions-in-action form the mental component internal to the action and cause the bodily movement. In this respect, focusing on higher hierarchical levels (e.g., mental images and intentions) to indirectly control lower motor levels (e.g., muscle patterns) seems to be more functional as compared to a direct control of movement. Recent studies have indeed shown that movement efficiency is enhanced by an external focus (Zachry et al., 2005; Marchant et al., 2009) as compared to directing attention to the body movements themselves (i.e., internal focus; Totsika and Wulf, 2003; Wulf et al., 2003, 2009; Wulf, 2007). When performing well-practiced acts, we actually do better when not thinking about the movements: “Keep your eye on the place aimed at, and your hand will fetch it; think of your hand, and you will very likely miss your aims.” (James, 1890). This effect clearly varies with the level of expertise of the performer. The expert has attentional resources available that can be directed away from the movement. In the present experiment, focusing on the intended effect – dynamicity – might have allowed the TC group the exploitation of unconscious or automatic processes (Wulf et al., 2001; Wulf and Lewthwaite, 2010), resulting in greater Motor Flexibility. Flexibility appears when willfulness disappears (Fraleigh, 2000).

## Conclusion

The purpose of this study was to investigate the effect of TC practice combined with MI on the movement kinematics of a reach-to-grasp task. This is unique in that no one has investigated this combination with quantitative kinematic data. The present results suggest that focusing on mental images is effective in increasing motor efficiency of daily actions (i.e., reach to grasp) in trained TC participants. In particular, this procedure ensures the necessary degree of accuracy and the lowest possible cost, as indexed by decreased wrist velocity and deceleration, and later closing of the hand while approaching the object. This, in turn, indicates the ability to carefully land the arm without the muscular effort of a sudden stop and to correctly place fingertips on the object without the need for a safety margin. Interestingly, adopting a natural image with a positive emotional connotation during MI produces a slowing down during the reaching phase – a measure associated with decreased mechanical effort, in both trained and untrained individuals. In terms of motor flexibility, the combination of TC and MI practice can develop the ability of activating and maintaining multiple motor plans, as highlighted by a greater trajectory deviation specific for the image with higher dynamic impact. In this regard, internal variability – indexed by the Range – appears to be a good predictor of expertise, therefore providing a fruitful tool to quantify the level of motor performance. Finally, MI training seems to help focus attention for the long term, as suggested by the CPT outcome.

One of the advantages of TC is that it’s a simple, useful practice that may promote motor control without special equipment. However, our study suggest that to achieve optimal benefits continued practice for an extended period of time is necessary.

Our paradigm, characterized by the adoption of new and not-overlearned images, could be useful to study the relation between mental processes and motor action in both trained and untrained populations. Movement kinematics can not only provide an accurate measure of the effect of MI on actions, but could also offer a novel tool for the diagnosis of potential deficits. Future research should seek to investigate the efficacy of MI and TC in situations where an individual’s motor function has been compromised, for example, following stroke or in patients with Parkinson’s Disease. Kinematics, however, is only an indirect measure of muscular effort. A new approach to properly assess Efficiency and Flexibility in the motor domain could be the integration of 3-D analysis of movement during EMG studies. This procedure might allow a deep investigation of muscle function and provide standardized indexes to be compared across different experiments.

Many questions remain to be addressed in future research, as the practice of TC has only recently started to gain broader attention in western countries. Knowledge about the unique biomechanical features of TC might better inform clinical decisions and further explicate the mechanisms of successful mind-body medicine.

## Data Availability Statement

All datasets generated for this study are included in the article/supplementary material.

## Author Contributions

ES and LS designed the study. AS analyzed the data. ES, AS, MG, and LS wrote the manuscript. LS critically revised the manuscript.

## Conflict of Interest

The authors declare that the research was conducted in the absence of any commercial or financial relationships that could be construed as a potential conflict of interest.
